# Tendencies in Operative Ideals

**Published:** 1918-09

**Authors:** A. H. Hipple

**Affiliations:** Omaha, Neb.


### TENDENCIES IN OPERATIVE IDEALS

BY A. H. HIPPLE, OMAHA, NEB.

There have been some tendencies in operative dentistry that are of doubtful advantage, in the main the tendencies are unquestionably upward.

There has been a complete change within the past few years in our ideas and our ideals as dentists. A few years ago we were chiefly concerned over physical problems; today our chief concern is in regard to vital problems. If you doubt that statement, compare the program of this meeting with the programs of national meetings held in former years. We are still interested in the restoration of lost dental tissue, and of lost dental organs, we are still engaged in the work of conservation, but we are more concerned today regarding the conservation of health than we are regarding the conservation of teeth. We do not know very much yet as to the relationship between dental conditions and systemic conditions, but we know something about it and we are anxious to learn more. There never was a time in the history of dentistry when dentists would spend as much time and money and travel as far to learns things as they will today. Dentists have had their vision enlarged. The dentistry of the past has been weighed in the balance and found wanting and we all realize it. In the past we have done the things we ought not to have done, and we have left undone the things we should have done, and we are all sorry for it. Within the past few years there have been times when our responsibilities have weighed so heavily upon some of us that we felt like giving up the practice of dentistry entirely. In his Special Dental Pathology Dr. G. V. Black, speaking of his own efforts to combat pyorrhea, says: ''I have since had reason to believe that I went much too far in my effort to cure. Much too large a percentage of those people are dead.'' Reflections of that character have a sobering influence, and we have all been doing more or less thinking along those lines. One result of our thinking has been a desire for knowledge, real definite knowledge, knowledge that will enable us to do better and safer dentistry, and that desire, in my opinion, is the most hopeful tendency in dentistry today.

The essayist refers to the tendency to substitute the gold inlay for the malleted gold filling, and silicate cements for baked porcelain fillings, as due partly to their merits, partly to a lack of skill on the part of many dentists, and partly to a desire on the part of patients for less trying operations, and more esthetic results. I agree with him in his analysis and in his conclusions. I agree with him that it is unfortunate that dentists in selecting filling materials should be guided by what is easiest, rather than by what is best. I deplore any lowering of standards. But, as I look over this country, as I study the results of examinations of mouths of school children, as I observe conditions that exist among army recruits, as I consider the foci of infection that we all know exist in the mouths of a very large percentage of our population, and interpret them in the light of hospital records and reports, I am impressed with the idea that in the very near future we will face the necessity of doing very much more work than we are now doing, and that we may be forced to adopt time-saving operations at the expense of some of our ideals. That is exactly what is being done at this very time in caring for the mouths of army recruits. The instructions are to fill occlusal cavities with amalgam, and proximal cavities with cement. That is not ideal dentistry by any means, but in the opinion of men in authority it is the best that can be done under the circumstances. It is better to fill all the cavities that need filling with cement and amalgam, than to spend the same amount of time in filling ten per cent. of the cavities with inlays or malleted gold fillings, and leave the rest unfilled. There are not enough dentists in the country today to do the work of the army and our civilian population that is imperatively demanded. For a few years at least, the shortage is bound to become more acute. The normal output of the dental colleges is being reduced, partly because of the adoption of the four-year course, and partly because of war conditions. In time the situation will be remedied and the supply of dentists will equal the demand, but in the meantime we will have to solve practical problems in a practical way.—*Journal N. D. A.*, August, 1918.

